# Simulation-Based Training for Therapeutic Relationship Competencies in Mental Health Nursing: A Cross-Sectional Evaluation

**DOI:** 10.3390/nursrep16050156

**Published:** 2026-05-06

**Authors:** María José Ferreira, Ana Laguía, Gabriela Topa

**Affiliations:** 1 Escuela Universitaria de Enfermería de Lugo, Centro Adscrito a la USC, 27002 Lugo, Spain; mariajose.ferreira@usc.es; 2 Department of Social and Organizational Psychology, Faculty of Psychology, Universidad Nacional de Educación a Distancia (UNED), 28040 Madrid, Spain; gtopa@psi.uned.es

**Keywords:** mental health nursing, therapeutic communication, standardised patient simulation, simulation-based learning, therapeutic relationship, experiential learning, nursing education research

## Abstract

**Background/Objectives**: Therapeutic communication is a core competency in mental health nursing, yet clinical placements often offer limited opportunities for undergraduate students to practise relational skills in a safe and structured way. Simulation, particularly when aligned with the Healthcare Simulation Standards of Best Practice™ (INACSL), may provide a useful context for fostering empathy, emotional presence, and professional communication. This study aimed to evaluate undergraduate nursing students’ satisfaction and self-confidence following participation in a standardised-patient simulation designed to address therapeutic relationship competencies. **Methods**: A descriptive cross-sectional study was conducted with 142 third-year nursing students at a public university. Participants completed two INACSL-aligned simulation encounters involving psychiatric scenarios that required therapeutic engagement. After the sessions, students completed a questionnaire based on the Student Satisfaction and Self-Confidence in Learning Scale, adapted to the context of the simulation. Data were analysed using descriptive statistics. **Results**: Students reported high levels of satisfaction and self-confidence following the simulation experience. Between 88.0% and 92.9% of participants agreed or strongly agreed with items related to realism, relevance, and motivation. High levels of agreement were also observed for items related to therapeutic communication, critical thinking (98.6%), clinical competence (95.8%), and teamwork (93.6%). Lower levels of agreement were found for the usefulness of video-based debriefing (61.9%) and the adequacy of material resources (57.1%), suggesting areas for improvement in future implementation. **Conclusions**: Standardised-patient simulation was positively evaluated by nursing students and was associated with high levels of satisfaction and self-confidence in learning. The findings suggest that this type of educational strategy may support students’ perceived development of therapeutic communication and relational skills in mental health nursing education. However, these results are based on self-reported data collected using an adapted measurement approach and should be interpreted with caution. Further research using validated instruments and performance-based measures is needed to assess competence development more directly.

## 1. Introduction

Therapeutic communication and the establishment of a trusting nurse–patient relationship are core competencies in mental health nursing and central to the quality of care provided to people experiencing psychological distress. Relational skills such as empathy, emotional attunement, active listening, and professional presence support patient engagement, reduce perceived stigma, and promote recovery-oriented care [1,2]. However, opportunities for undergraduate nursing students to practise these competencies in clinical environments remain limited. Ethical concerns, patient vulnerability, unpredictable symptom presentations, and constraints on supervision frequently restrict the extent to which learners can safely participate in real psychiatric encounters [3].

Simulation has emerged as a robust pedagogical approach capable of bridging this gap. It provides psychologically safe, structured, and clinically relevant environments where students can rehearse complex skills without exposing patients to risk [4,5,6,7], a framework originally conceptualised in seminal healthcare simulation research [8]. Expanding evidence shows that simulation-based learning has been associated with improvements in clinical reasoning, communication, and professional confidence across nursing specialties [9,10,11,12], while simultaneously reducing students’ stress and anxiety levels [13]. By navigating high-pressure scenarios within a controlled environment, students develop the necessary skills to manage complex clinical situations. This emotional mastery not only bolsters self-confidence in real-life settings but also fosters greater clinical comfort, thereby improving both workplace performance and patient safety.

In mental health contexts, standardised-patient simulations have demonstrated particular value in promoting authentic interpersonal engagement, which is especially relevant in psychiatric settings [14,15]. Since stigma toward individuals with mental health disorders is prevalent among nursing students [16,17,18,19], who often lack the confidence to communicate effectively with these patients, simulation training in this field is highly relevant. While several studies have demonstrated its success within mental health nursing curricula [19,20,21,22], further research remains necessary.

The recent Healthcare Simulation Standards of Best Practice™ [23] provide internationally recognised guidance for simulation design, facilitation, debriefing, and evaluation, emphasising learner-centredness, relational fidelity, and psychological safety. Although simulation research has expanded substantially in recent years, most published studies continue to prioritise acute care or technical scenarios. Comparatively fewer studies have examined simulation explicitly designed to address therapeutic relationship competencies in mental health nursing [3,24].

Therefore, further empirical evidence is needed to examine students’ perceptions of learning experiences derived from relationally focused simulation. The present study addresses this need by evaluating undergraduate nursing students’ satisfaction and self-confidence in learning following participation in a standardised-patient simulation designed in accordance with the Healthcare Simulation Standards of Best Practice™. Specifically, this study examines students’ perceptions of communication, critical thinking, teamwork, and self-confidence after participation in a relationally oriented psychiatric simulation. In doing so, this study contributes to the literature by focusing specifically on relational and therapeutic communication competencies within mental health simulation, an area that remains less frequently examined compared to technical or acute-care scenarios. Moreover, it also provides empirical evidence on students’ perceived learning experiences in this context.

From an educational perspective, this intervention can also be understood through established learning frameworks. Experiential learning theory suggests that knowledge is constructed through concrete experience followed by reflection [25,26,27], a process that is central to simulation-based education. In this context, simulation provides a structured opportunity for students to engage in emotionally complex interactions, while guided debriefing facilitates reflective practice and the integration of new insights into professional behaviour [28,29]. In addition, socio-constructivist perspectives emphasise that learning emerges through interaction, dialogue, and shared meaning-making within a psychologically safe environment. These factors are core elements of standardised-patient simulation and are consistent with current simulation standards [23]. These theoretical perspectives help explain the potential of simulation to support the development of relational competencies in mental health nursing, where communication is inherently contextual, interpersonal, and ethically sensitive. Accordingly, the present study is explicitly positioned as an educational evaluation. It focuses on students’ immediate perceived learning outcomes—specifically satisfaction and self-confidence in learning—rather than directly assessing objectively measured clinical competence [30]. This approach aligns the analytical scope with the exploratory nature of the study’s design. Within this framework, the simulation design in the present study specifically targets relational competence development through structured interaction, guided reflection, and progressive exposure to emotionally complex clinical scenarios.

## 2. Materials and Methods

### 2.1. Design

A descriptive cross-sectional study was conducted to evaluate undergraduate nursing students’ perceptions of a standardised-patient simulation designed to address therapeutic communication competencies in mental health settings. This study adhered to the STROBE guidelines for cross-sectional studies. A completed STROBE checklist with page numbers is provided as Supplementary Material. The study followed methodological recommendations for educational research in nursing, including clarity of aims, representativeness of the sample, and transparent reporting of procedures [31]. Simulation activities were designed and implemented in accordance with the Healthcare Simulation Standards of Best Practice™ [23], particularly the standards on Simulation Design, Facilitation, Professional Integrity, and Evaluation. These standards were operationalised through key principles including structured scenario design, facilitated debriefing, and an emphasis on psychological safety and learner-centred approaches.

The scenarios underwent faculty review and pilot testing to ensure consistency, clarity and relational fidelity before implementation. Pilot testing was conducted with a small group of volunteer senior nursing students (not included in the study sample) and two experienced mental health nursing faculty members. Feedback focused on scenario clarity, timing, emotional realism, and alignment with learning objectives. Minor refinements were made to scenario flow and facilitator prompts to enhance relational fidelity and ensure a psychologically safe learning environment.

### 2.2. Participants

A convenience sample of 142 third-year undergraduate nursing students enrolled in a Mental Health Nursing course at a European public university participated in the study. All students registered in the course were invited, resulting in a 100% response rate. Participants were 20–25 years old, and 86.6% identified as female—a demographic distribution consistent with national nursing education trends. Participation was voluntary, and no incentives were provided.

### 2.3. Simulation Intervention

Two simulation sessions were conducted in the university’s clinical simulation laboratory. Scenarios were developed by experienced mental health nursing faculty and aligned with the Healthcare Simulation Standards of Best Practice™. The scenarios used peer standardised patients—senior nursing students specifically trained to portray realistic psychiatric encounters through structured role preparation sessions, including guidance on case consistency, emotional expression, and interaction patterns to ensure a comparable level of standardisation across simulations.

The two simulation scenarios were designed to reflect common clinical situations in acute mental health care requiring therapeutic communication skills. Both scenarios involved adult patients experiencing emotional distress and were set in a psychiatric care context. While both focused on relational interaction, they differed in clinical presentation and communication demands. One scenario emphasised emotional containment and active listening in a patient presenting depressive symptoms, whereas the second scenario required students to manage more complex interpersonal dynamics, including anxiety, distress, and communication challenges. This variation was intended to expose students to different relational situations while maintaining a consistent level of complexity across the learning experience.

Prior to simulation, students received theoretical preparation on depression, emotional distress, therapeutic communication techniques, and the Reserved Therapeutic Space (*Espacio Terapéutico Reservado*, ETR) model, which emphasises relational presence in acute psychiatric care [32]. All students received the same theoretical preparation prior to the simulation sessions, ensuring consistency in instructional content across participants.

Each student directly participated in at least one scenario and engaged in structured observational learning during peers’ performances. Simulation sessions were conducted in small groups of approximately 25–30 students per session, with two faculty facilitators supervising each group. Each simulation session, including participation and observation phases, lasted approximately 50 min. Debriefing was conducted immediately after each simulation session using a facilitated reflective approach led by faculty members. Each debriefing lasted approximately 20–30 min and was facilitated by trained mental health nursing faculty. The debriefing process included three phases: (1) an initial reaction phase, allowing students to express immediate emotional responses; (2) an analysis phase, focusing on communication strategies, therapeutic interaction, and clinical reasoning; and (3) a summary phase, highlighting key learning points and linking the experience to clinical practice. Thus, the debriefing process focused on encouraging students to reflect on their communication strategies, emotional responses, and relational skills displayed during the interaction. Facilitators guided discussion to ensure a psychologically safe environment and to help students interpret their performance constructively. When available, video recordings of the simulation were optionally used to support reflection and discussion. Although video review was incorporated as a complementary tool, students reported heterogeneous perceptions of its usefulness, as reflected in the item-level distribution (Table 1).

### 2.4. Instrument

To evaluate students’ perceptions of the simulation experience, a set of self-report items was used to assess key learning-related outcomes, including satisfaction with the learning experience and self-confidence in learning during the standardised-patient simulation.

#### Student Satisfaction and Self-Confidence in Learning

Student satisfaction and self-confidence in learning were assessed using an ad hoc questionnaire developed drawing on items from the Student Satisfaction and Self-Confidence in Learning Scale (SCLS) [33,34]. The original SCLS was used as a conceptual reference to guide item formulation; however, several items were reworded and contextualised to reflect the specific characteristics of the standardised-patient simulation and the educational context in which it was conducted.

To support content validity, the adapted items were reviewed by experienced nursing faculty with expertise in simulation-based education. This process ensured the relevance, clarity, and alignment of the items with the learning objectives of the simulation.

The resulting 13-item instrument retained the two conceptual domains of the original scale: (1) satisfaction with the learning experience (5 items; e.g., “Watching recorded performances was useful”), and (2) self-confidence in learning (8 items; e.g., “The experience improved my clinical competence”). Items assessed students’ perceptions of the usefulness of the simulation, the adequacy of the teaching methods and materials, and their perceived development of knowledge and clinical skills through the standardised-patient simulation.

Participants rated each item using a Likert-type response scale indicating their level of agreement with the statements (1 = strongly disagree to 5 = strongly agree). Higher scores indicated greater satisfaction with the learning experience and higher perceived self-confidence in learning.

The internal consistency of the scale was assessed using Cronbach’s alpha. The reliability coefficient was 0.58 for the satisfaction dimension and 0.72 for the self-confidence dimension. The overall reliability of the full scale (13 items) was α = 0.80. The relatively low internal consistency observed for the satisfaction dimension may be related to the adaptation and contextualization of items, the limited number of items or heterogeneity in students’ interpretations. Given this limitation, results from the satisfaction dimension should be interpreted as exploratory indicators rather than as stable or psychometrically robust measurements. Accordingly, findings derived from this subscale are reported descriptively and should be considered with particular caution.

Regarding descriptive statistics, the first dimension showed a mean of 4.18 (SD = 0.44), whereas the second dimension presented a mean of 4.29 (SD = 0.39). Finally, the correlation between the two dimensions was r = 0.67, p < 0.001.

The questionnaire should be understood as a context-sensitive educational evaluation tool rather than as a formal validation of the original SCLS. The original scale was used as a conceptual reference to guide item formulation; however, items were adapted and contextualised to reflect the specific characteristics of the standardised-patient simulation and the relational learning objectives of the intervention. For this reason, the instrument is described as ad hoc, as it was designed to capture students’ perceptions within this specific educational context rather than to establish psychometric properties or to generalise findings beyond the study setting. Accordingly, the results are interpreted descriptively and with caution, particularly for the satisfaction dimension, whose internal consistency was below commonly accepted thresholds.

### 2.5. Procedure

Immediately following the simulation sessions, students completed the questionnaire anonymously using a secure online form (Microsoft Forms). Students were given dedicated time to complete it using their own devices. Participation was voluntary, no academic incentives were offered, and students were explicitly informed that their decision to participate in the study would not influence their academic evaluation. Data were collected in aggregate form. While the instrument was administered during a compulsory session, participation remained strictly voluntary. Students were informed of their right to decline participation, withdraw at any time without penalty, or submit incomplete questionnaires. As a result, the response rate was 100%, with no missing data observed. No identifying information or audiovisual recordings were collected or analysed. Data were exported into IBM SPSS Statistics version 29 for analysis.

Although the response rate was 100%, participation was voluntary and unrelated to academic evaluation. The questionnaire was administered immediately after the simulation during scheduled class time, which may have facilitated full participation. However, we acknowledge that collecting data in a structured academic context may introduce subtle participation pressure or socially desirable responding, and this possibility should be considered when interpreting the findings.

### 2.6. Data Analysis

The analytical strategy was intentionally descriptive, consistent with the exploratory and evaluative purpose of the study. Frequencies, means and standard deviations were calculated to summarise responses for each SCLS item. The objective was not to test causal hypotheses or compare subgroups, but to examine the overall distribution of student perceptions following a curricular simulation intervention implemented with a single cohort. Given the absence of comparison groups and the census-based inclusion of the entire class, inferential statistics were not considered methodologically appropriate. This approach aligns with recommendations for educational programme evaluation studies focused on implementation feedback and quality improvement.

## 3. Results

A total of 142 students completed the post-simulation questionnaire (response rate: 100%). Overall, students reported high levels of satisfaction and self-confidence following the standardised-patient simulation. Agreement (agree or strongly agree) with items related to satisfaction with the learning experience ranged from 88.0% to 92.9%, indicating positive perceptions of realism, relevance and motivation. High levels of agreement were observed for items related to critical thinking (98.6%), clinical competence (95.8%), teamwork (93.6%) and team communication (98.6%).

### 3.1. Satisfaction with the Simulation Experience

Most students evaluated the simulation positively. Scenario realism was rated highly (88.0% agreed or strongly agreed), and 91.6% reported that the activity was motivating and professionally relevant. Session duration was considered appropriate by 92.9% of participants.

Perceptions of material resources were less homogeneous: although 57.1% rated them as adequate, 23.9% were neutral and 19.0% expressed some level of dissatisfaction, indicating variability in perceived environmental fidelity, as shown in the item-level results (Table 1).

### 3.2. Perceived Development of Therapeutic and Clinical Competencies

Students reported high levels of agreement regarding items related to therapeutic communication and clinical competencies:

- Critical thinking: 98.6% agreed or strongly agreed the simulation supported clinical reasoning;
- Clinical competence: 95.8% reported increased self-confidence in applying skills;
- Teamwork: 93.6% reported improved collaborative abilities;
- Team communication: 98.6% agreed the activity supported team communication effectiveness.

Confidence in taking a leadership role was more variable (73.2% agreement), suggesting it may be a secondary benefit for learners at this stage of training.

### 3.3. Variability in Perceptions of Video-Based Debriefing

Less agreement was observed regarding the usefulness of reviewing recorded performances: only 61.9% rated video review as helpful, while 31.0% were neutral and 7.0% disagreed. These findings indicate different levels of comfort with self-observation and highlight opportunities to refine the emotional framing and structure of audiovisual debriefing, as reflected in students’ responses (Table 1).

### 3.4. Item-Level Findings

Item-level responses for all 13 items of the SCLS are presented in Table 1. Overall, results show consistently high agreement across both subscales—satisfaction with learning and self-confidence in learning. As expected, the strongest areas of agreement corresponded to items related to communication, critical thinking and clinical competence. Two items showed greater variability: the perceived usefulness of video-based debriefing and the adequacy of material resources. These areas indicate opportunities for enhancing environmental fidelity and structured audiovisual reflection in future implementations.

In addition to the response distributions, item-level descriptive statistics indicated generally high central tendency across most items, with mean scores ranging from 3.49 to 4.58. Lower mean values were observed for items related to material resources and video-based debriefing, suggesting comparatively less favourable evaluations in these areas.

Variability in responses was also greater for items related to video review, leadership roles, and material resources, as reflected in their wider distribution of responses. This pattern suggests that students’ perceptions were less homogeneous in these aspects of the simulation experience, potentially reflecting differences in individual comfort with self-observation, perceived role readiness, or expectations regarding the learning environment.

At the domain level, the positive correlation observed between satisfaction and self-confidence in learning (r = 0.67, p < 0.001) suggests that students who reported more favourable learning experiences also tended to perceive greater confidence in their learning, although this association should not be interpreted as indicative of causal or structural relationships.

**Table 1:** Student Responses to the Student Satisfaction and Self-Confidence in Learning Scale (*N* = 142).

Item	Mean (SD)	Strongly Agree	Agree	Neutral	Disagree	Strongly Disagree
Scenarios were realistic	3.99 (0.65)	15.5%	72.5%	7.0%	4.9%	0.0%
Cases reflected learning goals	4.46 (0.66)	51.4%	44.4%	1.4%	2.8%	0.0%
The experience was motivating	4.37 (0.69)	47.9%	43.7%	6.3%	2.1%	0.0%
Video review was useful	3.79 (0.91)	24.6%	37.3%	31.0%	6.3%	0.7%
Session length was appropriate	4.3 (0.61)	38.0%	54.9%	6.3%	0.7%	0.0%
Material resources were adequate	3.49 (0.95)	12.0%	45.1%	23.9%	18.3%	0.7%
Helped prioritise therapeutic actions	4.36 (0.58)	40.8%	54.2%	4.9%	0.0%	0.0%
Improved clinical competence (perceived)	4.42 (0.57)	45.8%	50.0%	4.2%	0.0%	0.0%
Enhanced teamwork	4.47 (0.68)	55.6%	38.0%	4.9%	0.7%	0.7%
Leadership role was necessary	3.89 (0.83)	22.5%	50.7%	19.7%	7.0%	0.0%
Improved team communication	4.58 (0.52)	59.2%	39.4%	1.4%	0.0%	0.0%
Strengthened critical thinking (perceived)	4.58 (0.52)	59.9%	38.7%	1.4%	0.0%	0.0%
Debriefing provided useful feedback	4.51 (0.53)	52.8%	45.8%	1.4%	0.0%	0.0%

Note. Percentages represent the proportion of students selecting each response option. Items correspond to the two subscales of the SCLS: satisfaction with learning (five items) and self-confidence in learning (eight items). Percentages are rounded to one decimal place; totals may not equal 100% due to rounding.

## 4. Discussion

The present study examined nursing students’ perceptions following participation in a standardised-patient simulation designed to address therapeutic communication in mental health settings. Overall, students reported high levels of satisfaction and self-confidence in learning, particularly in relation to communication, teamwork, and critical thinking. These findings should be interpreted as indicators of perceived learning and educational acceptability rather than as evidence of actual competence development.

The results are consistent with prior simulation research reporting high levels of learner satisfaction and perceived competence gains across nursing education contexts [9,14,35]. In contrast to many studies centred on acute or technical scenarios, the present intervention focused explicitly on therapeutic communication. The strong endorsement of communication- and teamwork-related items suggests that students valued the opportunity to rehearse relational interactions within a structured and psychologically safe learning environment.

However, interpretation of these findings must remain cautious. The data reflect self-reported perceptions collected immediately after the intervention and therefore represent perceived learning rather than objectively measured competence. Positive post-simulation evaluations are common in educational research and may be influenced by emotional engagement, novelty effects, or social desirability. Consequently, the present results should be understood as evidence of favourable learner appraisal rather than confirmation of improved clinical performance. In this study, the outcomes assessed should be understood as indirect proxies of therapeutic relationship competencies rather than direct measures of relational performance.

### 4.1. Variability in Debriefing and Resource Adequacy

Two aspects of the simulation experience showed greater variability: video-based debriefing and material resource adequacy. Although most students evaluated video review positively, a substantial proportion reported neutral or less favourable perceptions. Previous literature suggests that audiovisual debriefing may require careful facilitation and structured reflection to maximise its educational value. Variability in responses may therefore reflect differences in students’ comfort with self-observation or in the perceived structure of the feedback process.

Perceptions of material resources were also mixed. While more than half of participants considered the available resources adequate, a notable minority expressed dissatisfaction. Environmental and material conditions can influence immersion and perceived realism in simulation-based education. Addressing these elements in future implementations may enhance consistency in learner experience. In this context, material resources refer to the physical and audiovisual elements supporting the simulation environment, including room setup, equipment, and recording tools used during the sessions.

### 4.2. Limitations and Future Research

Several limitations should be acknowledged. First, the cross-sectional design precludes conclusions regarding long-term retention of learning or transfer to clinical settings. Second, reliance on self-report measures introduces the possibility of response bias and social desirability effects. Furthermore, although the questionnaire was developed drawing on items from the Student Satisfaction and Self-Confidence in Learning Scale (SCLS), several items were reworded and contextualised to better reflect the specific characteristics of the standardised-patient simulation and the educational setting of the study. Consequently, the instrument should be considered an ad hoc measure rather than a strict adaptation of the original scale. In addition, the internal consistency observed for the satisfaction dimension was relatively low (α = 0.58), which is likely related to the adaptation of items and their contextual specificity, and therefore results from this dimension should be interpreted with caution. Future research could benefit from further psychometric evaluation of the instrument or from the use of fully validated versions of the SCLS in comparable educational contexts. Third, the study was conducted in a single academic institution, limiting generalisability to other educational or cultural contexts. Finally, no observational or performance-based measures were included, restricting interpretation to subjective evaluations of learning outcomes.

Future research should incorporate longitudinal follow-up, direct performance-based assessments, and the use of observational rubrics or communication-specific measures capable of capturing therapeutic relationship behaviours more accurately. Furthermore, incorporating qualitative studies would allow for a deeper exploration of students’ perceptions regarding the learning acquired through simulation, providing insight into the subjective development of their clinical confidence. In addition, multi-institutional samples would help determine whether these perceived gains are sustained over time and to what extent they are reflected in observable clinical behaviours.

### 4.3. Educational Implications

Despite these limitations, the findings suggest that relationally focused simulation may represent a feasible and well-received educational strategy within undergraduate mental health nursing curricula. High levels of student satisfaction and perceived self-confidence indicate that such experiences are well received and considered relevant to professional preparation.

The variability observed in debriefing and resource-related items also provides practical guidance for programme refinement. Enhancing the structure of audiovisual debriefing and improving environmental conditions may strengthen the overall learning experience. Integrating simulation activities that explicitly target therapeutic communication can complement traditional clinical placements by offering structured opportunities to practise relational skills in a controlled setting.

## 5. Conclusions

Standardised-patient simulation was positively evaluated by undergraduate nursing students participating in a mental health training activity focused on therapeutic communication. High levels of satisfaction and perceived self-confidence were observed, particularly in relation to communication, clinical reasoning, and teamwork. Greater variability in perceptions of video-based debriefing and material resources highlights specific areas for improvement in future implementation. These findings reflect students’ perceived learning experiences rather than objective improvements in clinical competence. More specifically, satisfaction and perceived self-confidence were particularly associated with domains such as therapeutic communication, teamwork, and critical thinking, as reflected in the item-level results. While the results support the feasibility and educational acceptability of relationally focused simulation, further research using longitudinal designs and performance-based measures is needed to more accurately assess its impact on therapeutic communication competencies in clinical practice.

## Data Availability

The data supporting the findings of this study are not publicly available due to ethical and confidentiality considerations, as the dataset includes responses from nursing students participating in a clinical simulation-based educational intervention. Anonymised data are available from the corresponding author upon reasonable request for academic and research purposes. The decision not to deposit the dataset in a public repository was made to ensure participant confidentiality and compliance with institutional ethical approval conditions.

## Supplementary Materials

The following supporting information can be downloaded at: https://www.mdpi.com/article/10.3390/nursrep16050156/s1. A completed STROBE checklist with page numbers is provided as Supplementary Material.

## Public Involvement Statement

No public involvement in any aspect of this research.

## Guidelines and Standards Statement

This manuscript was drafted against the STROBE Statement for observational cross-sectional research.

## Use of Artificial Intelligence

To ensure linguistic precision, ChatGPT (Version 5.2 pro) was utilised solely for spelling, punctuation, and grammatical refinement. The complete document was processed through the platform to identify clarity improvements and phrasing suggestions, all of which were manually reviewed and curated before implementation. This tool was strictly used for editing purposes; no new content was generated, and the original meaning and intent of the text remained unaltered throughout the process. After using this service, the authors reviewed, edited, and accepted the suggested improvements as they deemed appropriate. The authors take full responsibility for the final content and accuracy of the manuscript.

## Abbreviation

The following abbreviation is used in this manuscript:

SCLS	Student Satisfaction and Self-Confidence in Learning Scale
