# Supplementary material for: Simulation-Based Training for Therapeutic Relationship Competencies in Mental Health Nursing: A Cross-Sectional Evaluation
Source: Nurs Rep. 2026 May 6;16(5):156. doi: 10.3390/nursrep16050156 (PMC13210155; doi:10.3390/nursrep16050156)
Supplement: Supplementary file 1 [file nursrep-16-00156-s001.zip › nursrep-4184512-Supplementary Material.pdf]

STROBE Checklist

Cross-sectional studies

Section	Item No.	Recommendation	Reported on page(s)
**Title and Abstract**	1a	Indicate the study's design with a commonly used term in the title or abstract	Title; Abstract (page 1)
	1b	Provide an informative and balanced summary of what was done and what was found	Abstract (page 1)
**Introduction**	2	Explain the scientific background and rationale for the investigation	Section 1 (Introduction; (pages 2–3)
	3	State specific objectives, including any prespecified hypotheses	Section 1 (Introduction; final paragraph – page 3)
**Methods**	4	Present key elements of study design early in the paper	Section 2.1 (Design; page 3)
	5	Describe the setting, locations, and relevant dates	Section 2.2 (Participants; page 3); Section 2.3 (Simulation Intervention; page 4)
	6a	Give eligibility criteria and sources/methods of participant selection	Section 2.2 (Participants; page 3)
	6b	For matched studies, give matching criteria and number of exposed/unexposed	Not applicable
	7	Clearly define all outcomes, exposures, predictors, confounders	Sections 3 (Results, pages 6–8)
	8	Give sources of data and details of assessment methods	Section 2.4 (Instrument; page 4); Section 2.5 (Procedure; page 5)
	9	Describe any efforts to address potential sources of bias	Section 4.2 (Limitations and Future Research; page 9)
	10	Explain how the study size was arrived at	Section 2.2 (Participants; page 3)
	11	Explain how quantitative variables were handled in the analyses	Section 2.6 (Data Analysis; page 6)
	12a	Describe all statistical methods, including those used to control for confounding	Section 2.6 (Data Analysis; page 6)
	12b	Describe any methods used to examine subgroups and interactions	Not applicable

STROBE Checklist

Cross-sectional studies

	12c	Explain how missing data were addressed	Section 2.2 (Participants, page 3; 100% response rate)
	12d	If applicable, describe analytical methods taking account of sampling strategy	Not applicable
	12e	Describe any sensitivity analyses	Not applicable
**Results**	13a	Report numbers of individuals at each stage of study	Section 3 (Results, pages 6–8)
	13b	Give reasons for non-participation at each stage	Section 2.2
	13c	Consider use of a flow diagram	Not applicable
	14a	Give characteristics of study participants	Section 2.2 (Participants, page 3)
	14b	Indicate number of participants with missing data for each variable	Section 2.2 (Participants, page 3; 100% response rate)
	15	Report numbers of outcome events or summary measures	Section 3 (Results – Table 1, page 7)
	16a	Give unadjusted estimates and, if applicable, confounder-adjusted estimates	Section 3 (Results – Table 1, page 7–8)
	16b	Report category boundaries when continuous variables were categorized	Not applicable
	16c	If relevant, translate estimates of relative risk into absolute risk	Not applicable
**Discussion**	17	Summarise key results with reference to study objectives	Section 4 (Discussion, page 8)
	18	Discuss limitations of the study	Section 4.2 (Limitations and Future Research; page 9)
	19	Give a cautious overall interpretation of results	Section 4 (Discussion, page 8)
	20	Discuss generalisability (external validity) of the study results	Section 4.1 (Variability in Debriefing and Resource Adequacy; page 8)
**Other Information**	21	Give the source of funding and role of funders	Funding Statement (page 10)
